# Tobacco and Alcohol on Television: A Content Analysis of Male Adolescents’ Favorite Shows

**DOI:** 10.5888/pcd15.180062

**Published:** 2018-11-01

**Authors:** Brittney Keller-Hamilton, Jacqueline Muff, Traci Blue, Bo Lu, Michael D. Slater, Megan E. Roberts, Amy K. Ferketich

**Affiliations:** 1 The Ohio State University College of Public Health, Columbus, Ohio; 2 The Ohio State University School of Communication, Columbus, Ohio

## Abstract

**Introduction:**

Media tobacco and alcohol portrayals encourage adolescent substance use. Preventing adolescent initiation with these substances is critical, as they contribute to leading causes of morbidity and mortality in the United States. Television tobacco and alcohol portrayals have not been examined for more than 7 years. This study analyzed tobacco and alcohol portrayals on adolescents’ favorite television shows and evaluated the rate of portrayals by parental rating.

**Methods:**

Adolescent males (N = 1,220) from Ohio reported 3 favorite television shows and how frequently they watch them. For each of the 20 most-watched shows in the sample, 9 episodes were randomly selected and coded for visual and verbal tobacco and alcohol incidents. Demographics of characters who used or interacted with the substances were recorded. Negative binomial regression modeled rates of tobacco and alcohol incidents per hour by parental rating.

**Results:**

There were 49 tobacco and 756 alcohol portrayals across 180 episodes. Characters using the products were mostly white, male, and adult. The rate of tobacco incidents per hour was 1.2 for shows rated TV-14 (95% CI, 0.4–3.6) and 1.1 for shows rated TV-MA (95% CI, 0.3–4.5). The estimated rate of alcohol incidents per hour was 20.9 for shows rated TV-14 (95% CI, 6.3–69.2) and 7.2 for shows rated TV-MA (95% CI, 1.5–34.1).

**Conclusions:**

Adolescent males’ favorite television shows rated TV-14 expose them to approximately 1 tobacco incident and 21 alcohol incidents per hour on average. Limiting tobacco and alcohol incidents on television could reduce adolescents’ risk of substance use.

## Introduction

Tobacco and alcohol use contribute substantially to the leading causes of morbidity and mortality in the United States (1,2). Adolescence presents an important window for preventing adult misuse of these products, as most adult tobacco users initiated use during adolescence (3) and alcohol misuse and dependence in adulthood are associated with alcohol use in adolescence (4). Although recent decreases have been observed in the prevalence of current combustible tobacco (5) and alcohol (6) use among adolescents, there is room for improvement. Decreases in combustible tobacco product use were offset by increases in electronic cigarette use (5) and current alcohol use among high school students had a prevalence of 32.8% in 2015 (6).

Exposure to media portrayals of tobacco and alcohol use leads to increased risk of adolescent tobacco and alcohol use (7–9). Whereas a substantial body of research has quantified the amount of tobacco and alcohol in movies, few studies have examined tobacco and alcohol portrayals on television. Although no studies have been conducted on the tobacco or alcohol content of television shows airing in the past 7 years, previous research indicates that tobacco and alcohol portrayals are numerous on broadcast television programs popular among adolescents (10,11). Moreover, parental ratings were not particularly useful in predicting whether a show contained tobacco or alcohol portrayals (10,11).

Adolescents in the United States watch approximately 2 hours of television per day, and the increased use of streaming services facilitates watching shows that no longer air, or never aired, on broadcast television (12). The lack of recent analysis of adolescents’ favorite shows, across all television platforms, is an important gap in the literature. The primary objective of this study was to describe the frequency of tobacco and alcohol portrayals on adolescents’ favorite television shows. A secondary objective was to examine whether the rates of tobacco and alcohol portrayals differed by parental rating.

## Methods

### Study population and design

Male adolescents aged 11 to 16 years (N = 1,220), from 1 urban and 9 Appalachian Ohio counties, were recruited into the Buckeye Teen Health Study between January 2015 and June 2016. Most subjects were recruited via address-based sampling (n = 991); the remainder were recruited via convenience sampling (n = 229). The survey-weighted mean age of study participants was 13.9 years (standard error = 0.06) and 68.2% were white non-Hispanic. Because an aim of the larger study (which includes boys only) was to identify predictors of smokeless tobacco use, girls were excluded. Additional details about the study procedures are provided elsewhere (13). This study was approved by The Ohio State University’s institutional review board.

Trained interviewers asked youth to list their 3 favorite television shows and recorded programs. For each show, interviewers asked participants to recall how many episodes they watched in a typical week. Participants were asked not to list sporting events or sports programs, the news, movies on television, or special television events (eg, awards shows). A total of 1,068 (87.5%) participants listed at least 1 favorite show. Shows were weighted by the number of episodes participants watched in a typical week, and the 20 most-watched shows were selected for analysis. First, a sample of 3 seasons of each television show was randomly selected; seasons were excluded if any episodes within them first aired after January 1, 2015. This exclusion assured that we did not sample any episodes that aired after we started data collection. Next, 3 episodes were randomly selected from each season for content analysis, resulting in 9 total episodes analyzed from each show. Five shows had aired fewer than 3 seasons before 2015. In these cases, 9 episodes that had aired before 2015 were randomly selected. One television show (*Doctor Who*) had been airing since 1963; in this case, we excluded episodes that first aired before the show’s 2005 reboot.

### Television show coding procedures

A codebook and coding procedures were designed on the basis of prior television content analysis studies (11,14). The codebook was extensively pilot tested and revised through multiple iterations by the authors. Four undergraduate students were trained on coding procedures, which involved double-coding episodes, comparing results, and settling on final coding decisions in rotating pairs such that each student coded 6 episodes of every show.

The final coding instrument captured visual and verbal portrayals of tobacco and alcohol. All types of tobacco products (ie, cigarettes, electronic cigarettes, smokeless tobacco, cigars or cigarillos, hookah, pipes, or dissolvable tobacco) and alcoholic beverages were counted. Visual mentions were only counted once per character per scene, unless the character got a new product. For example, a group of 4 characters drinking beer in a bar would be coded as 4 instances of visual alcohol use regardless of how many times they drank from their glasses. If 1 character were to order and start drinking a second beer, a fifth instance would be added. Verbal mentions were also recorded as character- and scene-specific counts per episode. Thus, if 2 characters were to have a prolonged conversation about alcohol in one scene, it was coded as 1 verbal instance per character (as opposed to coding every single time alcohol is referenced by either character). If the characters resumed their conversation later in another scene, it was coded as 2 more verbal instances.

For visual portrayals, incidents were further classified as visual use (a character or crowd of characters using the product); visual nonuse (a character or crowd of characters interacting with the product but not using it, such as a bartender serving a drink or a character holding a drink but never drinking it); visual impairment (a character or crowd of characters observed clearly under the influence of the product, but not seen using the product [ie, hungover or drunk characters]); background portrayals (eg, bottles of wine on a kitchen counter); and visual cues to use the products (eg, ashtrays or empty wine glasses). Additionally, for all visual incidents, we recorded the associated character’s sex (male, female, inconclusive, and mixed-sex crowd), race/ethnicity (non-Hispanic white, non-Hispanic black, Hispanic, Asian, mix of multiple race/ethnicities in a crowd, and other/unknown), and age (child or adolescent, young adult, adult, elderly, unknown, and crowd of mixed ages). Finally, we recorded whether visual use of the product was peer motivated (yes/no) and whether the character using the product was in an obviously positive/happy mood (yes/no).

### Statistical analyses

We calculated descriptive statistics for characteristics of visual tobacco and alcohol incidents. Because we had no hypotheses related to the gender, age, race/ethnicity, peer motivation, or positive mood of tobacco or alcohol users on the television shows, hypothesis testing was not conducted.

We next estimated rates of visual and verbal tobacco and alcohol portrayals per hour (ie, not per episode) by using product-specific negative binomial regression models. The dependent variable was the sum of verbal and visual tobacco and alcohol incidents across all 9 episodes of each show. The offset term in the models was the log of the sum of minutes per show, summed across all 9 episodes. Episode length did not include commercials, and the range of episode lengths in our sample was 10 to 76 minutes. In models estimating the rate of tobacco and alcohol portrayals across all episodes, intercepts were exponentiated and multiplied by 60 to estimate rates per hour. Finally, we again used negative binomial regression to compare rates of visual and verbal tobacco and alcohol incidents by parental rating: TV-MA (mature audience only), TV-14 (parents strongly cautioned), and TV-PG/TV-Y7 (parental guidance suggested and directed to older children, respectively). Because there were zero tobacco or alcohol portrayals in shows rated TV-Y7, these shows were combined with the shows rated TV-PG to achieve stable estimates. The α for significance tests comparing rates of incidents by parental rating was .05. Analyses were completed with Stata version 14.2 (StataCorp).

## Results

Adolescents listed 623 unique television shows. The list of sampled television shows, parental ratings of each show, and the number of visual and verbal tobacco and alcohol incidents summed across 9 episodes are shown in Table 1.

**Table 1:** Portrayal of Tobacco and Alcohol on Male Adolescents’ Most-Watched Television Shows^a^, 10 Ohio Counties, 2015–2016

Parental Rating	Television Show	Popularity Ranking^b^	Percent of Youth Who Listed Show as a Favorite	Number of Tobacco Incidents^c^	Number of Alcohol Incidents^c^
**TV-Y7**					
	*The Amazing World of Gumball*	5	3.9	0	0
	*Gravity Falls*	15	2.7	0	0
**TV-PG**					
	*SpongeBob SquarePants*	1	10.8	1	0
	*The Flash*	6	5.1	0	63
	*Teen Titans Go*	8	3.0	0	0
	*The Regular Show*	9	3.6	0	0
	*Adventure Time*	10	3.5	0	2
	*Modern Family*	11	3.5	2	48
	*The Office*	12	3.1	0	40
	*Doctor Who*	14	3.4	1	41
	*Ridiculousness*	17	2.6	2	27
	*The Middle*	20	2.5	5	41
**TV-14**					
	*Family Guy*	3	8.0	9	103
	*The Big Bang Theory*	4	5.5	0	87
	*American Dad*	7	3.3	5	107
	*Impractical Jokers*	16	2.4	5	10
	*Arrow*	18	3.0	1	68
**TV-MA**					
	*The Walking Dead*	2	11.3	0	11
	*South Park*	13	2.9	5	32
	*Breaking Bad*	19	3.1	13	76

a Up to three favorite television shows were listed by adolescent males enrolled in the Buckeye Teen Health Study between January 2015 and June 2016. Shows were weighted by how many episodes adolescents reported watching in a typical week; the 20 most-watched shows were selected.

b Popularity ranking represents the frequency of how often the show was watched by adolescents in the study. For example, *SpongeBob SquarePants *was the most-watched show in our sample.

c Number of incidents includes verbal and visual product portrayals, summed across all 9 randomly selected episodes of television shows.

### Characteristics of visual tobacco and alcohol portrayals

Most tobacco and alcohol portrayals were visual (Table 2). For visual tobacco portrayals, most incidents (89.3%) involved a character using the product. These characters were nearly all male, and most were white non-Hispanic adults. There were no cases of peer-motivated tobacco use, and characters had an obviously positive/happy mood in 14.3% of tobacco incidents.

**Table 2:** Characteristics of 49 Tobacco and 756 Alcohol Portrayals Across 180 Episodes^a^, 10 Ohio Counties, 2015–2016

Characteristic	Tobacco Portrayals^b^ n (%)^c^	Alcohol Portrayals n (%)^c^
**Type of portrayal**		
Visual	36 (73.5)	534 (70.6)
Verbal	13 (26.5)	222 (29.4)
**Type of visual portrayal^d^**		
Individual	28 (77.8)	312 (58.4)
Crowd	0	49 (9.2)
Background	5 (13.9)	134 (25.1)
Visual cue	3 (8.3)	39 (7.3)
**Type of visual use/nonuse^e^**		
Use of product	25 (89.3)	147 (40.7)
Interaction with product, no use	3 (10.7)	201 (55.7)
Impairment, no use	0	13 (3.6)
**Sex of character^e^**		
Female	1 (3.6)	84 (23.3)
Male	27 (96.4)	241 (66.8)
Unknown/inconclusive	0	4 (1.1)
Mixed-sex crowd	0	32 (8.9)
**Race/ethnicity of character^e^**		
White non-Hispanic	18 (64.3)	268 (74.2)
Black non-Hispanic	2 (7.1)	22 (6.1)
Hispanic	5 (17.9)	9 (2.5)
Asian	0	18 (5.0)
Multiple race/ethnicities in crowd	0	26 (7.2)
Other/unknown	3 (10.7)	18 (5.0)
**Age of character^e^**		
Child/adolescent	1 (3.6)	9 (2.5)
Young adult	2 (7.1)	10 (2.8)
Adult	22 (78.6)	326 (90.3)
Elderly	1 (3.6)	0
Multiple ages in crowd	0	1 (0.3)
Unknown/inconclusive	2 (7.1)	15 (4.2)
**Motivation of character^e^**		
Peer motivated	0	15 (4.2)
Not peer motivated	28 (100.0)	346 (95.8)
**Mood of character^e^**		
Positive/happy mood	4 (14.3)	58 (16.1)
Neutral/negative mood	24 (85.7)	303 (83.9)

a Three episodes from 3 seasons of each show were randomly selected for analysis. All episodes aired before January 1, 2015.

b Tobacco portrayals include portrayals of any tobacco product (ie, cigarettes, electronic cigarettes, smokeless tobacco, cigars or cigarillos, hookah, pipes, or dissolvable tobacco).

c Percentages may not sum to 100 due to rounding.

d This item was only coded for visual portrayals.

e These items were only coded for individual or crowd visual portrayal types.

For visual alcohol portrayals, a majority were instances of a character interacting with a product but not using it (55.7%). Like tobacco portrayals, most characters involved in visual alcohol portrayals were white non-Hispanic, male, and adult. Few cases of alcohol use were peer-motivated (4.2%) or associated with an obviously positive/happy mood (16.1%).

### Rates of tobacco and alcohol portrayals per hour

A total of 49 visual and verbal tobacco portrayals appeared in our sample. Across all ratings, there was approximately 1 tobacco incident every 2 hours (Table 3). After conditioning on parental rating, shows rated TV-14 had more tobacco incidents per hour than shows rated TV-PG/TV-Y7 (rate ratio [RR] = 4.2; 95% confidence interval [CI], 1.003–17.7). The rate of tobacco incidents per hour for shows rated TV-MA did not differ from shows rated TV-14 (RR = 0.9; 95% CI, 0.2–5.7) or shows rated TV-PG/TV-Y7 (RR = 3.9; 95% CI, 0.7–21.0).

**Table 3:** Rates of Tobacco and Alcohol Portrayals Per Hour of Television^a^, 10 Ohio Counties, 2015–2016

Category	Tobacco Portrayals Rate (95% CI)	Alcohol Portrayals Rate (95% CI)
Overall rate per hour	0.6 (0.3–1.3)	9.6 (5.0–18.7)
**Rate per hour by parental rating^b^**		
TV-PG and TV-Y7	0.3 (0.1–0.7)	5.5 (2.5–12.1)
TV-14	1.2 (0.4–3.6)	20.9 (6.3–69.2)
TV-MA	1.1 (0.3–4.5)	7.2 (1.5–34.1)

Abbreviation: CI, confidence interval.

a A negative binomial regression was used to estimate rates of combined visual and verbal tobacco and alcohol portrayals during 60 min of television (excluding commercials).

b Rate ratios cited in the text are based on unrounded values and may differ from rate ratios calculated from this table, which were rounded values.

There were 756 visual and verbal alcohol portrayals in our sample of television shows, resulting in nearly 10 alcohol incidents per hour (Table 3). Rates of alcohol incidents did not differ by parental rating (results not shown), though the RR comparing the rate of alcohol incidents in shows rated TV-14 to shows rated TV-PG/TV-Y7 was marginally significant (RR = 3.8; 95% CI, 0.9–15.7).

## Discussion

Television shows watched by adolescent males contained approximately 1 tobacco portrayal every 2 hours and 10 alcohol portrayals every hour. Characters who used or interacted with these products were largely white non-Hispanic, male, and adult. Television shows rated TV-14 had a higher rate of tobacco portrayals per hour than shows rated TV-PG/TV-Y7, but the rate did not differ from shows rated TV-MA. Our results are consistent with prior literature in finding that both substances are prevalent on television shows watched by adolescents, and that there is little evidence of different rates of substance portrayals according to parental rating (10,11).

With adolescents watching approximately 2 hours of television per day (12), the frequency of tobacco and alcohol portrayals is concerning. Cultivation theory (15) suggests that by shaping how they perceive the real world, frequent portrayals of tobacco and alcohol use on television may influence adolescents’ health behaviors. Indeed, a substantial body of research has demonstrated that more (versus less) exposure to tobacco or alcohol in movies is associated with increased risk of adolescent use of those products (7–9). Further, social cognitive theory (16) describes the powerful effects that media portrayals of substance use may have on adolescents’ health behaviors by teaching them about these behaviors through role models. Again, the literature supports this assertion, at least for tobacco use, by demonstrating that adolescents whose favorite movie stars smoke cigarettes are more likely to be cigarette smokers themselves (17,18).

It is unsurprising that most of the tobacco and alcohol portrayals in our sample of shows were by white non-Hispanic male characters, as our participants were all male and most were white non-Hispanic. One’s media selections are related to gender and social identity, and thus our television shows largely had characters who looked like our participants (19). Additionally, the reinforcing spirals approach suggests that people select media content that portrays behaviors in which they are interested or engage, and that these selected media exposures in turn may reinforce these interests or behaviors (20). In our study, this approach would imply that youth who watch shows with high rates of tobacco or alcohol portrayals may be especially interested in these behaviors, and that watching such portrayals reinforces their interest in these substances. Furthermore, adolescent males who identify strongly with characters on television are more likely to learn from the behaviors modeled by those characters (21,22). Thus, the fact that our subjects were largely watching characters who looked like them portraying tobacco and alcohol use suggests that these exposures may be particularly persuasive.

One way to reduce adolescents’ exposure to tobacco and alcohol portrayals on television would be to require that shows carry a TV-MA rating if they depict substance use. Currently, substance use is not considered when assigning television ratings in the United States (23). With parents giving increasing attention to the ratings of shows their children are allowed to watch (24), this presents an opportunity to markedly reduce youth exposure to substance use portrayals on television.

In addition to strengthening parental ratings, interventions to address how adolescents react to portrayals of substances on television may prove effective in reducing adolescent substance use. Potential interventions could involve improving adolescents’ self-control and media literacy. By reducing positive expectancies of product use, good self-control modifies the effect of movie exposure to tobacco and alcohol on adolescent substance use behaviors (25), and interventions targeting self-control have been successful with other outcomes (26). Similarly, studies testing media literacy interventions have found them to be successful in both increasing adolescents’ media literacy and reducing their susceptibility to smoking (27–29).

Our study had several strengths. First, rather than relying on current broadcast ratings to sample television shows, we sampled shows based on what adolescents actually watched, and how often they watched them. This resulted in our coding shows that adolescents continue to watch even after the series ends (eg, *The Office*). Additionally, shows appeared in our sample that air on cable and are not included in broadcast ratings (eg, *SpongeBob SquarePants*). Together, this approach led to a more valid estimation of the rate of tobacco and alcohol portrayals adolescent males are exposed to per hour of television watching than would have been achieved through older methods of television show sampling.

The following limitations should be considered when interpreting our study results. First, the present results pertain to adolescent males in Ohio and may not generalize to other samples. Although the shows reported by the youth in this study are also popular (or were at one time popular) nationwide, it is possible that regional preferences drove the sample of television shows. Indeed, although 11 of the top 20 shows in our sample were the most-watched among both urban and rural participants in our study, re-drawing the sample of television shows after stratifying on urban/rural status would have led to different samples of shows. Similarly, a sample of adolescent girls would likely produce a somewhat different sample of shows displaying potentially different rates of tobacco and alcohol use. Second, because several hundred unique shows were listed by study youth, it was not feasible to analyze the content of each show; therefore, we were unable to estimate how exposure to tobacco and alcohol use on television is associated with adolescent use of these products in our study. Third, our coding scheme did not collect specific information about the type of tobacco product used, and thus we cannot describe whether the balance of tobacco products portrayed on television is similar to the balance of products used among adolescents. Finally, we observed substantial heterogeneity in rates of tobacco and alcohol portrayals across shows with the same parental rating in our sample; for example, the range of alcohol portrayals across 9 episodes of shows rated TV-14 was 10 to 107 portrayals. This heterogeneity contributed to large confidence intervals for our estimated rate ratios. It is possible that this is due in part to sampling only 9 episodes from each show. Future studies analyzing more episodes within each TV rating category could result in greater precision of estimated rates.

In conclusion, adolescents are exposed to high rates of tobacco and alcohol portrayals on television. Though rated as acceptable for underage youth (ie, aged 14 years and older), shows rated TV-14 depict at least 1 tobacco incident and nearly 21 alcohol incidents per hour on average. Evidence from the movie literature suggests that greater exposure to substance use in the media places adolescents at increased risk of tobacco and alcohol initiation (7–9). Future research should examine this association as it pertains to television exposures to inform interventions aiming to reduce adolescent tobacco and alcohol initiation. Consideration of substance portrayal on television shows when assigning parental ratings may help reduce adolescent tobacco and alcohol use.

## References

[1] US Department of Health and Human Services. The health consequences of smoking — 50 years of progress: a report of the Surgeon General. Rockville (MD); 2014.

[2] Johnson NB , Hayes LD , Brown K , Hoo EC , Ethier KA ; Centers for Disease Control and Prevention (CDC). CDC National Health Report: leading causes of morbidity and mortality and associated behavioral risk and protective factors—United States, 2005-2013. MMWR Suppl 2014;63(4):3–27. 25356673

[3] US Department of Health and Human Services. Preventing tobacco use among youth and young adults. A report of the Surgeon General. 2014.

[4] McCambridge J , McAlaney J , Rowe R . Adult consequences of late adolescent alcohol consumption: a systematic review of cohort studies. PLoS Med 2011;8(2):e1000413. 10.1371/journal.pmed.1000413 21346802 PMC3035611

[5] Jamal A , Gentzke A , Hu SS , Cullen KA , Apelberg BJ , Homa DM , Tobacco use among middle and high school students - United States, 2011-2016. MMWR Morb Mortal Wkly Rep 2017;66(23):597–603. 10.15585/mmwr.mm6623a1 28617771 PMC5657845

[6] Esser MB , Clayton H , Demissie Z , Kanny D , Brewer RD . Current and binge drinking among high school students - United States, 1991-2015. MMWR Morb Mortal Wkly Rep 2017;66(18):474–8. 10.15585/mmwr.mm6618a4 28493857 PMC5657986

[7] Dal Cin S , Stoolmiller M , Sargent JD . When movies matter: exposure to smoking in movies and changes in smoking behavior. J Health Commun 2012;17(1):76–89. 10.1080/10810730.2011.585697 22085232 PMC3252424

[8] Hanewinkel R , Sargent JD . Longitudinal study of exposure to entertainment media and alcohol use among German adolescents. Pediatrics 2009;123(3):989–95. 10.1542/peds.2008-1465 19255030 PMC2746499

[9] Waylen A , Leary S , Ness A , Sargent J . Alcohol use in films and adolescent alcohol use. Pediatrics 2015;135(5):851–8. 10.1542/peds.2014-2978 25869367 PMC8194465

[10] Cullen J , Sokol NA , Slawek D , Allen JA , Vallone D , Healton C . Depictions of tobacco use in 2007 broadcast television programming popular among US youth. Arch Pediatr Adolesc Med 2011;165(2):147–51. 10.1001/archpediatrics.2010.276 21300655

[11] Eisenberg ME , Larson NI , Gollust SE , Neumark-Sztainer D . What are we drinking? Beverages shown in adolescents’ favorite television shows. J Acad Nutr Diet 2017;117(5):763–9. 10.1016/j.jand.2016.12.004 28185861 PMC5409859

[12] The Nielsen Company. The Nielsen total audience report: Q1 2017. 2017.

[13] Friedman KL , Roberts ME , Keller-Hamilton B , Yates KA , Paskett ED , Berman ML , Attitudes toward tobacco, alcohol, and non-alcoholic beverage advertisement themes among adolescent boys. Subst Use Misuse 2018;53(10):1706–14. 10.1080/10826084.2018.1429473 29436898 PMC6037539

[14] Eisenberg ME , Larson NI , Gollust SE , Neumark-Sztainer D . Snacking on television: a content analysis of adolescents’ favorite shows. Prev Chronic Dis 2016;13:E66. 10.5888/pcd13.160014 27197079 PMC4877182

[15] Gerbner G . Toward “cultural indicators”: the analysis of mass mediated public message systems. AV Commun Rev 1969;17(2):137–48.

[16] Bandura A . Social cognitive theory of mass communication. Media Psychol 2001;3(3):265–99. 10.1207/S1532785XMEP0303_03

[17] Distefan JM , Gilpin EA , Sargent JD , Pierce JP . Do movie stars encourage adolescents to start smoking? Evidence from California. Prev Med 1999;28(1):1–11. 10.1006/pmed.1998.0409 9973581

[18] Tickle JJ , Sargent JD , Dalton MA , Beach ML , Heatherton TF . Favourite movie stars, their tobacco use in contemporary movies, and its association with adolescent smoking. Tob Control 2001;10(1):16–22. 10.1136/tc.10.1.16 11226355 PMC1763998

[19] Zillmann D , Bryant J . Selective exposure to communication. Mahwah (NJ): Lawrence Erlbaum Associates, Inc; 1985.

[20] Slater MD . Reinforcing spirals: the mutual influence of media selectivity and media effects and their impact on individual behavior and social identity. Commun Theory 2007;17(3):281–303. 10.1111/j.1468-2885.2007.00296.x

[21] Huesmann LR , Lagerspetz K , Eron LD . Intervening variables in the TV violence-aggression relation: evidence from two countries. Dev Psychol 1984;20(5):746–75. 10.1037/0012-1649.20.5.746

[22] Paik H , Comstock G . The effects of television violence on antisocial behavior: a meta-analysis. Communic Res 1994;21(4):516–46. 10.1177/009365094021004004

[23] Federal Communications Commission. Commission finds industry video programming rating system acceptable; adopts technical requirements to enable blocking of video programming (the “V-chip”) [news release]. Washington (DC); 1998.

[24] Hart Research Associates. Key findings from 2014 TV ratings research among parents. Washington (DC); 2014.

[25] Wills TA , Gibbons FX , Sargent JD , Gerrard M , Lee HR , Dal Cin S . Good self-control moderates the effect of mass media on adolescent tobacco and alcohol use: tests with studies of children and adolescents. Health Psychol 2010;29(5):539–49. 10.1037/a0020818 20836609 PMC3719172

[26] van Genugten L , Dusseldorp E , Massey EK , van Empelen P . Effective self-regulation change techniques to promote mental wellbeing among adolescents: a meta-analysis. Health Psychol Rev 2017;11(1):53–71. 10.1080/17437199.2016.1252934 27796160

[27] Pinkleton BE , Weintraub Austin E , Cohen M , Miller A , Fitzgerald E . A statewide evaluation of the effectiveness of media literacy training to prevent tobacco use among adolescents. Health Commun 2007;21(1):23–34. 10.1080/10410230701283306 17461749

[28] Primack BA , Douglas EL , Land SR , Miller E , Fine MJ . Comparison of media literacy and usual education to prevent tobacco use: a cluster-randomized trial. J Sch Health 2014;84(2):106–15. 10.1111/josh.12130 25099425 PMC4126196

[29] Shensa A , Phelps-Tschang J , Miller E , Primack BA . A randomized crossover study of web-based media literacy to prevent smoking. Health Educ Res 2016;31(1):48–59. 26675176 10.1093/her/cyv062 PMC4883031

